# Acid-Base disorders as predictors of early outcomes in major Trauma in a resource limited setting: An observational prospective study

**DOI:** 10.11604/pamj.2014.17.2.2007

**Published:** 2014-01-06

**Authors:** Asiimwe Ian Shane, Wangoda Robert, Kwizera Arthur, Makobore Patson, Galukande Moses

**Affiliations:** 1 Hoima Regional Referral Hospital, Uganda; 2 Accident and Emergency Department, Mulago National Referral Hospital, Uganda; 3 Department of Anaesthesia, Makerere University, Uganda; 4 Department of Surgery, Makerere University, Uganda

**Keywords:** Major trauma, anion gap, early outcome, organ failure, acidosis

## Abstract

**Introduction:**

Mortality from trauma remains a major challenge despite recent substantial improvements in acute trauma care. In trauma care patient resuscitation to correct hypotension from volume loss still majorly relies on use of physiological parameters such as blood pressure, pulse rate, respiratory rate, urine output and oxygen saturation. In resource limited settings these methods may not be sufficient to detect occult tissue hypoxia and the accompanying metabolic derangements.

**Methods:**

A prospective observational study carried out at a level I urban Trauma centre; Accident and Emergency unit. Major trauma patients were consecutively recruited into the study. Venous blood samples were drawn for analysis of serum electrolytes, serum PH and anion gap. The venous blood gas findings were correlated with patients’ clinical outcome at two weeks. Ethical approval was obtained.

**Results:**

Ninety three major trauma patients were recruited, patients’ age ranged from 12 months to 50 years. Forty nine patients (53%) were acidotic (PH less than 7.32), 39 patients (42%) had low bicarbonate (bicarbonate level less than 21 mmol), 54 patients (58%) had high corrected anion gap (anion gap corrected of 16 or more). Fourteen patients (15%) developed secondary organ failure and 32 (34%) patients died.

**Conclusion:**

Metabolic acidosis is common among major trauma patients, its severity may be related to delay in initiating care. Acid base derangements were predictors of mortality among major trauma patients in this resource limited setting.

## Introduction

Mortality from trauma still remains a major challenge despite substantial improvement in acute trauma care. Trauma patient resuscitation to correct hypotension from volume loss still majorly relies on use of physiological parameters such as blood pressure, pulse rate, respiratory rate, urine output, Glasgow coma scale and oxygen saturation in low resource settings. This makes it difficult to detect occult tissue hypoxia, due to occult hypotension. Tissue hypoxia frequently results in significant metabolic acidosis. The resultant cellular and organ dysfunction can increase morbidity (length of hospital stay), and mortality. Serum pH, lactate, base deficit and bicarbonate have all been extensively studied as clinical markers of metabolic acidosis in shock however these studies have largely utilized arterial blood sample [1–4]. In this study, venous blood samples were used. In low resource countries there is limited data on pre-hospital care [5–8] and its implications on acid base balance and survival among major trauma.

The purpose of this study was to determine the acid-base derangements among emergency major trauma patients and clinical outcomes.

## Methods

**Study Design:** An observational, prospective study.

**Study Setting:** The study was carried out between January and April 2012 at Mulago National Referral Hospital located in Kampala, abustling Capital city of Uganda with a day time population of three million people. Mulago National Referral Hospital has a 1500 bed capacity, with an Accident and Emergency Unit (A & E) unit, an emergency ward (3BES), three general surgical units, a neuro surgical unit, and a paediatric surgical ward. It also has a cardiothoracic and orthopedic units and a Radiology unit. It is a level I trauma centre. The hospital also has a 15 bed intensive care unit, with a limited number of ventilators.

**Study participants** All trauma patients attended to at Mulago National referral hospital who met the eligibility criteria.

**Selection Criteria:** All age groups who presented to the A and E unit, with a history of trauma not exceeding 24 hour were recruited into the study Those patients who had received more than 500 ml of intravenous fluids were excluded.

**Sampling Procedure:** Purposive (convenience) sampling using inclusion and exclusion criteria was used in recruiting study participants.

**Sample Size Estimation:** The sample was 93 patients. Power of 90% and significance of 5% with estimate of 10% loss to follow up were factors considered.

### Data collection

Trauma patients were seen in A & E unit at or soon after admission into the hospital. Venous blood sample and vital history were taken (using a questionnaire) during primary survey and resuscitation and details were obtained after stabilization. The venous blood was obtained from the cubital veins for the adults and femoral veins when the cubital vein was inaccessible in children.

The patients were followed up to determine their early clinical outcomes measured as organ failure using Knaus score and mortality. The organs systems assessed during the follow up period were the cardiovascular, respiratory, renal and central nervous systems.

**Study variables** The predictor variables were serum PH, adjusted anion gap and Serum bicarbonate. Outcome variables were development of Organ failure and in hospital mortality during the study period.

Other study variables were; revised trauma score (RTS), age, pre-hospital time delay and sex.

### Data Analysis

Statistical analysis to establish the relationship between their initial acid base levels and adjusted anion gap with their early clinical outcome was done.

Data collected was entered into a computer and analyzed using SPSS software version 17.0. Data was summarized in form of proportions, frequency tables for categorical variables. Mean and standard deviation were used to summarize continuous variables. Scatter diagrams and box plots were also used to summarize results. Level of significance that acid base derangements are associated with secondary organ failure and mortality, determined using P-values.

### Quality control

Data from laboratory profiling was obtained on arrival at the emergency department was from samples drawn before fluid resuscitation, thus reflecting physiologic derangement before significant intervention. The blood samples were analyzed within 30 minutes of drawing, from the same automated chemistry analyzer machine, ABL FLEX835, to ensure coherent results. The laboratory was blinded to the state of the patients.

### Ethical considerations

Permission was obtained from the research and ethics committee of the College of Health Sciences at Makerere University Informed consent was obtained from the patient or their next of kin.

## Results

Of the 93 patients recruited in the study, age ranged from 12 months to 50 years. The mean age was 26.1 years. Forty nine (52.7%) patients were acidotic (PH less than 7.32), 39 (41.9%) patients had low bicarbonate (bicarbonate level less than 21mmol), 54 (58.1%) patients had high corrected anion gap (anion gap corrected of 16 or more). We had 14 (15.1%) patients who developed secondary organ failure and 32 (34.4%) patients died Table 1.

The time lag from the time of trauma and when the blood sample was taken off for analysis ranged from 1.0 hours to 12 hours. There were 55 (59.1%) patients who had their samples drawn at least 3 hours from the time of trauma, and 68 (75%) of the samples were drawn within 6 hours. The mean time for drawing samples was 4.65 hours and most patients arrived with in 2 hours (21.5%) followed by 1 hour (18.3%).

Serum PH levels of Major trauma patients were normally distributed about the reference line (7.32), the lower normal level for venous serum PH. Forty nine (52.7%) patients had serum PH <7.32 (NR 7.32-36).

The lowest measured value of serum bicarbonate was 3.8meq, and the highest measured level 29.9mmol (NR 21-29). A big proportion of major trauma patients 39 (41.9%) had serum PH of less than 21mmol Table 2.

**Figure 1:**
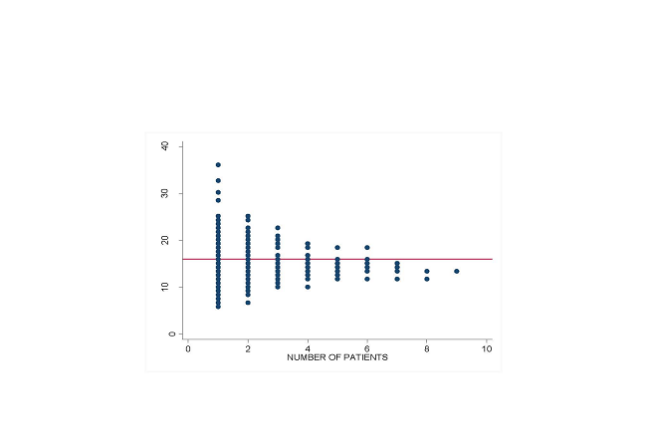
Corrected Anion gap levels of major trauma patients attended to at Mulago A & E unit

Thirty nine patients (41.9%) of the major trauma patients had serum anion gap corrected above 16. The lowest anion gap was 6.15 and the highest 35.75 (Figure 1).

**Figure 2:**
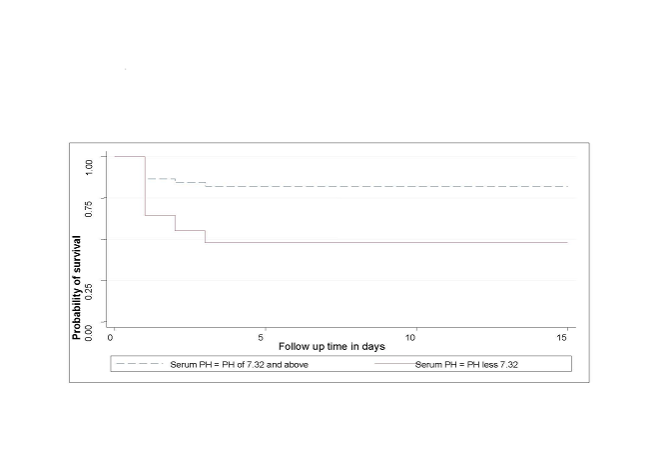
Kaplan-Meier survival estimates for patients with venous serum PH levels below and above 7.32

Those patients that had low initial serum PH of less than 7.32 were more likely to die and in bigger numbers than those with PH of 7.32 and above. This difference in survival between those that had PH < 7.32 and those that had PH >3.2 was statistically significant (log rank11.8, DF1 and P = 0.0006) (Figure 2).

## Discussion

This study set out to investigate the extent to which acid base derangements among major trauma patients occur at presentation at a tertiary hospital in a resource limited environment and how this relates to survival.

We found that acid base derangement (metabolic acidosis) (with venous PH of less than 7.32) was frequent among major trauma patients and all who died had significant acid base derangements. A big proportion of these trauma patients had high anion gap metabolic acidosis type.

The presence of metabolic acidosis in major trauma patients can be explained by increased production of organic acids however in trauma there is reduced clearance of these organic acids responsible for the clearance of organic acids. The chemical buffer system, most important the HCO_3_-/CO_2_ (bicarbonate, carbon dioxide) is consumed, resulting into low serum bicarbonate and carbon dioxide partial pressures a primary reduction in serum bicarbonate (HCO_3_-) concentration. Metabolic acidosis is a secondary decrease in the arterial partial pressure of carbon dioxide (PaCO2) and a reduction in blood PH [9] are additional occurrences. None of the study patients had other causes of metabolic acidosis. Causes other than trauma include uremia, ketoacidosis, and lactic acidosis.

In this study 38.7% of patients presented with primary organ failure and this was mainly; neurological (head injury), neurological with respiratory failure (head injury and blunt chest trauma) or respiratory failure (blunt chest and penetrating chest injury). Only 16.1% developed organ failure during the follow up and this agrees with findings of Ciesla [12]. Respiratory failure was the most common type in the follow up period which agrees with previous studies on trauma patients in which respiratory dysfunction was found to precede other organ dysfunctions[10, 15, 16]. Development of multi-organ failure following trauma has been reported to range from 7% to 75% with the majority developing respiratory failure [10–14]. Organ failure is associated with acid base derangements. There was a difference in means of serum PH, serum bicarbonate and anion gap corrected, among those that developed organ failure and those that did not, thought it was satisfactorily non-significant. However, according to previous studies using gastric PH a significant relationship occurs between organ dysfunction and low serum PH [2]. Perhaps our sample size was too small to show that the difference (only 14 patients developed secondary organ failure).

Patients with lower PH are more likely to die even in intensive care settings [3, 4, 17]. In this study we find that those who died had significantly lower serum PH, mean 7.257±0.114 compared with survivors mean PH 7.312±0.125 (p = 0.04), significantly lower bicarbonate 19.45±5.32 compared to survivors 21.8±4.46, (p = 0.04), and significantly higher anion gap corrected 18.35±5.99 compared to 14.97±5.1, p = 0.005. This agrees with studies that have been done using arterial blood gas analysis.

Surviving the first 24 hours following injury is associated with better outcome as most (about 70% of major trauma patients’ death occurred in the first 24 hours).

Most patients were brought by police ‘pickups’ as the medical ambulance system is not well developed in Uganda and the police did most of the transportation of trauma victims. There is limited pre-hospital care; but also upon arrival in hospitals there may be patient transfer delays, in triage, and in initiating active resuscitation. The average time from time of trauma incidence to time of drawing the blood sample was 4.65 hours. The time taken to draw the sample in this case reflects the time it takes for the trauma victim to access primary survey and resuscitation from the time of incident. All these may contribute to increase in proportion of patients with acid base derangements.

This study was without limitation; due to the varying patients to staff ratios during day and night may have which resulted in, both resuscitation and definitive treatment delays. These delays could have impacted on outcomes.

Only a limited major trauma patients had access to ICU, this may have increased the mortality among these major trauma victims. Blood samples for acid base derangements were drawn once and at the beginning, so the point at which correction of acidotic states occurs if it does was unknown.

## Conclusion

Metabolic acidosis was common among major trauma patients and impacted on survival.

**Table 1:** Summarizes patient demographic characteristics and morbidity and mortality at day 14. Acid base disorder trauma study, 2012

Demographic characteristic		(N)	%
**Sex**	Male	75	80.65
	Female	18	17.35
**Occupation**	Cyclists	29	31.2
	Students	15	15.1
	Businessmen and women	14	16.1
	Unemployed	13	14
	Others	22	23.7
**Mode of trauma**	Road traffic crush	60	64.5
	Assault	32	34.4
	Others	1	1.1
**Mode of arrival**	Police Ambulance	13	13.98
	Other Ambulances	8	8.6
	Police Pick-Up	56	60.22
	Others / Private Means	16	17.2
**Anatomical region injured**	Head and Neck	74	79
	Face	14	15.1
	Chest	15	16.1
	Abdomen	8	8.6
	Limbs	23	24.7
	External	62	66.7
**Patient disposition**	Discharged	50	53.8%
	Still In Hospital	9	9.7%
	Died^b^	32	34.4%
	Run away	2	2.2%
**Developed primary Organ dysfunction**	Neurological	22	23.7%
	Respiratory	4	4.3%
	Neurorespiratory	10	10.8%
	None	57	61.3%
**Development of secondary organ failure**	Developed Secondary Organ Failure	15^a^	16.13%
	No Development of Secondary Organ Failure	78	83.87%

a Most patients developed secondary respiratory dysfunction.

b The majority 22 (68.8%) died within the first 24 hours from the time of injury, the rest died on the second, 4 (12.5%), the third, 4 (12.5%) and fourth days, 2 (6.2%). There were no deaths beyond the fourth day.

**Table 2:** Acid base derangements, secondary organ failure mortality and survival among major trauma patients

Means	Developed organ failure	Did not develop	P-value
**Mean serum PH**	7.249±0.028	7.300±0.014	0.15
**Mean serum Bicarbonate**	18.9±4.8	21.8±4.5	0.07
**Mean Anion gap corrected**	19.5±4.3	14.9±5.1	0.94
**Patient variables**	Died (n = 32) survived	(n = 61)	
**Inpatient days (days)**	1.6 1±1	7.6±4.8	0.001
**Pre-hospital Time (hours)**	3.5±3.4	9.4±18.4	0.081
**Age (years)**	26.4±8.3	25.9±10.3	0.826
**ISS**	33.8±12.2	23.8±6.2	0.001
**RTS**	5.31±1.05	6.686±1.035	0.001
**Serum PH**	7.257±0.114	7.312±0.125	0.04
**Serum bicarbonate (MMol/l)**	19.45 ±5.32	21.8±4.46	0.020
**Serum albumin (g/dl)**	35.56±10.01	40.48±6.37	0.018
**Potasium (mmol/L)**	4.54±2.26	4.12±0.72	0.53
**Sodium (mmol/L)**	141.4±6.5	140.2±4.6	0.52
**Chloride (mmol/L)**	109.9±5.3	108.6±5.5	0.449
**Anion gap**	16.38±5.67	13.84±5.01	0.026
**Anion gap corrected**	18.35±5.99	14.97±5.1	0.005

## References

[1] Arnold TD, Miller M, van Wessem KP, Evans JA, Balogh ZJ (2011). Base deficit from the first peripheral venous sample: a surrogate for arterial base deficit in the trauma bay. J Trauma..

[2] Calvete JO, Schonhorst L, Moura DM, Friedman G (2008). Acid-base disarrangement and gastric intramucosal acidosis predict outcome from major trauma. Rev Assoc Med Bras..

[3] Kaplan LJ, Kellum JA (2008). Comparison of acid-base models for prediction of hospital mortality after trauma. Shock..

[4] Falcone RE, Santanello SA, Schulz MA, Monk J, Satiani B, Carey LC (1993). Correlation of metabolic acidosis with outcome following injury and its value as a scoring tool. World J Surg..

[5] Demyttenaere SV, Nansamba C, Nganwa A, Mutto M, Lett R, Razek T (2009). Injury in Kampala, Uganda: 6 years later. Can J Surg..

[6] Jayaraman S, Mabweijano JR, Lipnick MS, Caldwell N, Miyamoto J, Wangoda R (2009). Current patterns of prehospital trauma care in Kampala, Uganda and the feasibility of a lay-first-responder training program. World J Surg..

[7] Jayaraman S, Ozgediz D, Miyamoto J, Caldwell N, Lipnick MS, Mijumbi C (2011). Disparities in injury mortality between Uganda and the United States: comparative analysis of a neglected disease. World J Surg..

[8] Hsia RY, Ozgediz D, Mutto M, Jayaraman S, Kyamanywa P, Kobusingye OC (2010). Epidemiology of injuries presenting to the national hospital in Kampala, Uganda: implications for research and policy. Int J Emerg Med..

[9] Guyton AC, Hall JE (2006). The body fluids and the kidneys: text book of medical physiology eleventh edition.

[10] Ciesla DJ, Moore EE, Johnson JL, Burch JM, Cothren CC, Sauaia A (2005). The role of the lung in postinjury multiple organ failure. Surgery..

[11] Ciesla DJ, Moore EE, Johnson JL, Burch JM, Cothren CC, Sauaia A (2005). A 12-year prospective study of postinjury multiple organ failure: has anything changed. Arch Surg..

[12] Ciesla DJ, Moore EE, Johnson JL, Sauaia A, Cothren CC, Moore JB (2004). Multiple organ dysfunction during resuscitation is not postinjury multiple organ failure. Arch Surg..

[13] Botha AJ, Moore FA, Moore EE, Peterson VM, Goode AW (1997). Base deficit after major trauma directly relates to neutrophil CD11b expression: a proposed mechanism of shock-induced organ injury. Intensive Care Med..

[14] Kirton OC, Windsor J, Wedderburn R, Hudson-Civetta J, Shatz DV, Mataragas NR (1998). Failure of splanchnic resuscitation in the acutely injured trauma patient correlates with multiple organ system failure and length of stay in the ICU. Chest..

[15] Durham RM, Moran JJ, Mazuski JE, Shapiro MJ, Baue AE, Flint LM (2003). Multiple organ failure in trauma patients. J Trauma..

[16] Gerd Regel MG, Tobias Weltner, Johannes A (1996). Sturm, Hararld Tscherne. patterns of organ failure following severe trauma. World Journal of surgery..

[17] Kaplan LJ, Kellum JA (2004). Initial pH, base deficit, lactate, anion gap, strong ion difference, and strong ion gap predict outcome from major vascular injury. Crit Care Med..

